# PGE2 Promotes the Migration of Mesenchymal Stem Cells through the Activation of FAK and ERK1/2 Pathway

**DOI:** 10.1155/2017/8178643

**Published:** 2017-05-28

**Authors:** Xiaomin Lu, Jibin Han, Xiuping Xu, Jingyuan Xu, Ling Liu, Yingzi Huang, Yi Yang, Haibo Qiu

**Affiliations:** ^1^Department of Critical Care Medicine, Zhongda Hospital, Southeast University School of Medicine, No. 87, Ding Jiaqiao, Nanjing, 210009 Jiangsu, China; ^2^Department of Respiratory Medicine, Affiliated Jiangsu Province Hospital of Traditional Chinese Medicine, Nanjing University of Traditional Chinese Medicine, Nanjing, Jiangsu, China

## Abstract

A critical step of MSCs therapy is dependent on its ability to migrate into the sites of injury, so various approaches have been introduced to boost the migratory ability of MSCs. PGE2 is the major prostaglandin generated by COX enzymes and has been implicated in inflammatory response. Evidence indicates that PGE2 can facilitate MSCs migration. Further exploration of the underlying molecular mechanism participating in the promigratory ability of PGE2 may provide a novel strategy to improve MSC transplantation efficacy. In this study, our findings suggested that EP2 prostanoid receptor promotes MSCs migration through activation of FAK and ERK1/2 pathways. Furthermore, MSCs migration induced by PGE2 was blunted by FAK or ERK1/2 inhibitors. EP2-mediated MSCs migration depends on the activation of FAK and ERK1/2. However, the current study did not investigate the migration of MSCs over a blood vessel endothelial barrier. In conclusion, our findings reveal EP2-mediated FAK and ERK1/2 activation was essential for MSCs migration induced by PGE2, indicating that activation of EP2 receptor and FAK/ERK pathways may be a promising strategy to accelerate homing efficiency of MSCs, which in turn enhances therapeutic potential of MSCs transplantation.

## 1. Introduction

Mesenchymal stem cells (MSCs) are pluripotent nonhematopoietic cells derived from multiple sources, including bone marrow, adipose tissue, and umbilical cord blood [1–3]. Owing to their immunoregulatory, anti-inflammatory and proregenerative properties, MSCs are becoming an appealing candidate in treating various diseases [4, 5]. However, the therapeutic efficacy of MSCs is contingent upon their migratory capacity to sites of injury [6, 7]. Given only a small fraction of transplanted cells engrafted into injured tissue after MSC injection, various approaches have been introduced to facilitate engrafting efficiency, such as overexpression of chemokine receptor CXCR4 [8], or MSC cultured under hypoxic condition [9], both resulting in a higher retention of transplanted MSCs compared with control, offering incremental benefits.

PGE2 is the major prostaglandin generated by COX-1and COX-2 enzymes and exerts distinct actions in a broad array of physiologic and pathologic settings [10, 11]. PGE2 synthesis is markedly increased in the inflammatory setting [12]. It is well documented that PGE2 mediates both pro- and anti-inflammatory responses via binding to its four receptors, namely EP1-EP4 [13]. Aside from its role in inflammatory response, PGE2 also participates in proliferation and migration in several cell types [14–16]. In addition, Yun and his colleagues suggested that MSC migration can be boosted by PGE2 stimulation [17]. Among the four receptor subtypes, EP2 receptor activation was identified to link PGE2-induced MSC migration.

Focal adhesion kinase (FAK), also known as cell adhesion kinase, was initially identified in 1992 as a member of nonreceptor cytoplasmic tyrosine kinase [18]. The structure of FAK comprises an N-terminal region, a C-terminal region, and a central kinase region [19]. The N-terminal region of FAK serves as a linker between membrane integrin receptors and various growth factor receptors, transducing extracellular signaling input to cellular cytoskeleton. The C-terminal domain of FAK containing a ~100 sequence so called FAT and two proline-rich regions. FAT mediates binding with integrin-binding protein paxillin and talin, facilitating the adhesion of FAK to focal contact [20].

Over the last decades, accumulating evidence indicates that FAK functions in promoting cell migration in diverse array of normal and tumor cells [21, 22]. In addition, it has been proposed that the underlying mechanism FAK facilitated cell migration was through its promotion of cytoskeletal rearrangements and focal contact formation. Using fibroblast cell line, researchers demonstrated that FAK-deficient cells migrate poorly in response to growth factor stimulation [23]. Conversely, re-expression of FAK rescues the motility defect of fibroblast cell. Additionally, overexpression of FAK in ovary cells exhibited accelerated cell motility. Taken together, considering FAK signaling implicated in cell motility in various different cell lines, its function in the migration of MSCs is of potential importance.

## 2. Methods

### 2.1. Cell Line

Bone marrow-derived MSCs were purchased from Cyagen Biosciences (Guangzhou, China). CD45−, CD73+, CD90+, and CD105+ MSCs were confirmed by flow cytometry as we previously described [24]. We also evaluated the osteogenic differentiation and adipogenic differentiation capacity of MSCs using specific differentiation media. In our study, MSCs were obtained from one donor and undergone culture expansion in vitro to purify and generate sufficient numbers for transduction and experiment.

### 2.2. MSCs Culture

MSCs were seeded at 1 × 10^5^ cells/cm^2^ in DMEM containing 10% fetal bovine serum (FBS), 100 U/mL penicillin, and 100 *μ*g/mL streptomycin. MSCs were cultured at 37°C in a humidified atmosphere in an incubator with 5% CO_2_. Media were changed every 3 days. Adherent MSCs were washed with phosphate-buffered saline (PBS) and harvested by trypsinization with 0.25% trypsin. MSCs on the fourth to sixth passages were used for experiments.

### 2.3. Construction of MSCs Overexpressing EP2 Receptor

The EP2 plasmid was constructed as described previously [24]. Then, human embryonic kidney 293T cells (Cell Bank of the Chinese Academy of Sciences, Shanghai, China) were transfected recombinant lentiviral vectors with EP2 or GFP. MSCs from passages 4 to 6 were transduced with high-titer recombinant lentiviral vectors with EP2 or GFP. Because MSCs expressed both the EP2 and GFP genes (EP2-MSCs), the transduction efficiency was assessed with a fluorescence microscope. EP2 expression was assessed using Western blotting and PCR, as we described previously [24]. After MSCs were successfully transduced with recombinant EP2 plasmids, we evaluated cell surface markers (CD73, CD90, CD105, and CD45) and the osteogenic differentiation ability of MSC-EP2. In addition, we evaluated the migratory ability of MSCs via transwell assay following the completion of EP2 overexpression.

### 2.4. Migration Assay

MSCs migratory ability was assessed by a 24-well transwell system inserting a 8 *μ*m pore size filter membrane. 2 × 10^4^ cells in a serum-free medium of 200 *μ*L volume were added to the upper chamber, and 600 *μ*L serum-free medium or PGE2 (1 *μ*mol/L) was added to the bottom chamber. Subsequently, MSCs were incubated at 37°C for 6 h in serum free medium with PGE2 in the presence or absence of FAK inhibitor PF573228 (10 *μ*mol/L) or ERK1/2 inhibitor PD98059 (10 *μ*mol/L). 600 *μ*L serum-free medium in the presence or absence of PGE2 was added to the bottom chamber. After 6 h incubation at 37°C, the cells on the upper surface of the filter membrane were removed, whereas the migrated cells on the undersurface of the filter membrane were fixed with 4% paraformaldehyde, and cells on the undersurface of the filter were subsequently stained in crystal violet. Cells migrated to the lower surface of the filter were imaged using a microscope. Five fields from each filter were randomly chosen to determine the number of migrated cells.

### 2.5. Wound Healing Migration Assay

Migration was also evaluated by a wound healing migration assay. MSCs were cultured in 6-well plates until reached a confluent monolayer. Monolayers of MSCs were scratched with a disposable pipette tip and then wells were washed with phosphate buffered saline to remove the cell debris. Subsequently, MSCs were incubated at 37°C for 24 h in serum-free medium with PGE2 in the presence or absence of FAK inhibitor PF573228 (10 *μ*mol/L) or ERK1/2 inhibitor PD98059 (10 *μ*mol/L). The scratched area was photographed with a digital camera attached to a light microscope. Means were taken from five fields in the scratched area of each group. Migration assay was performed 3 separate times.

### 2.6. Western Blot Analysis

For Western blot, total protein from the cultured cells were extracted with RIPA reagents and stored at −80°C. Equal amounts of protein were electrophoresed on a 8% or 5% SDS-PAGE, then transferred to polyvinylidene fluoride membrane. Subsequently, membranes were blocked with TTBS (Tris-buffered saline with 0.1% Tween 20) containing 5% dry milk for 1 h at room temperature. After washing, PVDF membranes were incubated with indicated primary antibody for overnight at 4°C, followed by incubation with horseradish peroxidase-conjugated goat antirabbit IgG for 1 h at room temperature. Immunoreactive proteins were detected using a chemiluminescence kit. Band intensities were quantified using ImageJ software. Each experiment was performed 3 separate times.

### 2.7. Statistical Analysis

Data were presented as mean ± standard deviation (SD). Comparisons between two groups were made using an unpaired *t*-test. Differences among groups were analyzed by one-way analysis of variance (ANOVA) with Bonferroni's correction. The SPSS software 17.0 (SPSS, Chicago, IL, USA) was used for all statistical analyses. A *P* value of less than 0.05 was considered to be statistically significant.

## 3. Results

### 3.1. Characterization of MSCs

The cultured MSCs presented a uniformly spindle shape. MSCs were demonstrated to differentiate into adipocytes and osteocytes. MSCs exhibited very high expression of CD105, CD73, and CD90 but negligible expression of CD45 as previously reported [24].

### 3.2. MSCs Were Successfully Transduced with Recombinant EP2 Plasmids

Seven days after transduction, 97% of MSCs-EP2 were GFP positive, indicating the MSCs were stably transduced with lentivirus expressing either GFP or both GFP and EP2. The expression of GFP in the MSCs was detected under fluorescence also confirmed that MSCs were successfully transduced with recombinant EP2 plasmids. The Western blots showed that EP2 protein and mRNA expression in the EP2-MSCs was higher than that in the MSCs that had only received vector (MSC-vec) and the control MSCs, the detailed results reported in our published paper [24].

### 3.3. EP2 Overexpression Does Not Affect MSCs Surface Marker and Differentiation Ability

**Figure 1:**
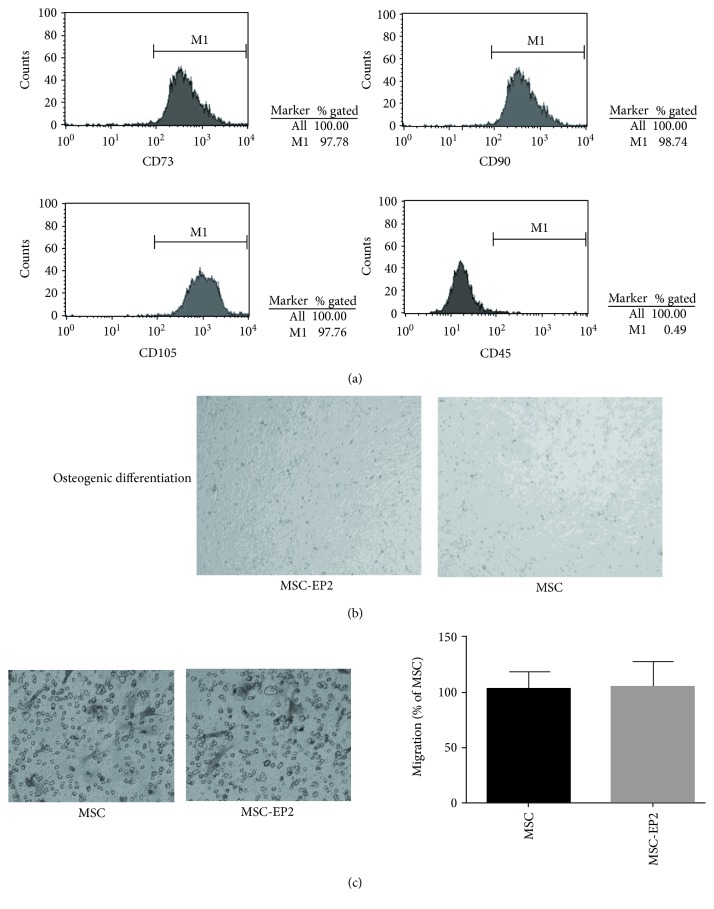
(a) The MSCs-EP2 were positive for CD73, CD90, and CD105 and negative for CD45. (b) Overexpression of EP2 does not impact MSCs osteogenic differentiation capability. (c) Transwell assay demonstrated that EP2 overexpression does not affect MSC migration, *P* > 0.05 (MSC versus MSC-EP2).

To explore the impact that over-expression of EP2 has on the functional characteristics of MSCs, we evaluated cell surface markers (CD73, CD 90, CD105, and CD45) of MSC-EP2 via flow cytometry. Furthermore, we evaluated the osteogenic and adipogenic differentiation ability of MSCs following the completion of EP2 overexpression. The MSCs-EP2 were positive for CD73, CD90, and CD105 and negative for CD45 (Figure 1(a)), suggesting that EP2 overexpression does not change cell surface receptor phenotype. In addition, overexpression of EP2 does not impact MSCs osteogenic differentiation capability (Figure 1(b)).

### 3.4. EP2 Overexpression Does Not Affect MSC Migration

To investigate whether EP2 overexpression affect MSC migration, we evaluated the migratory ability of MSCs via transwell assay following the completion of EP2 overexpression and found that EP2 overexpression alone did not affect MSCs migration (Figure 1(c)).

### 3.5. PGE2 Promotes Migration of MSCs

**Figure 2:**
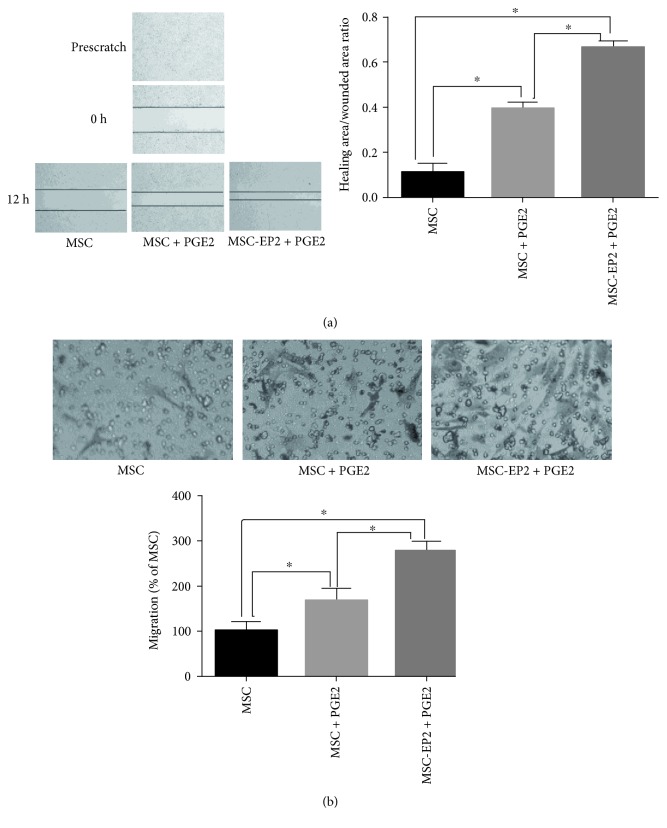
(a) Wound-healing assay demonstrated PGE2 facilitated MSCs and MSCs-EP2 migration. (b) Transwell assay demonstrated that PGE2 facilitated MSCs and MSCs-EP2 migration, ^∗^*P* < 0.05.

To examine whether PGE2 plays a role in the migration of MSCs, confluent MSCs were wounded and incubated for additional 24 h in serum-free medium with or without PGE2 (1 *μ*mol/L). In contrast to control, PGE2 evoked a substantial migration of the cells into the scratched area. Transwell filters were also used to further evaluate the migratory ability of MSCs stimulated with or without PGE2 (1 *μ*mol/L); PGE2-treated MSCs almost filled the scratched area and the width distance was significantly narrower than that of MSCs without PGE2 stimulation (Figure 2(a)). To clarify the activation of EP2 receptor which may underlie PGE2-induced MSCs migration. Transwell assay revealed that the number of MSCs-EP2 traversing the membrane was significantly more than the control when stimulated with PGE2 (Figure 2(b)).

### 3.6. FAK Signaling Contributes to PGE2-Mediated Migration of MSCs

**Figure 3:**
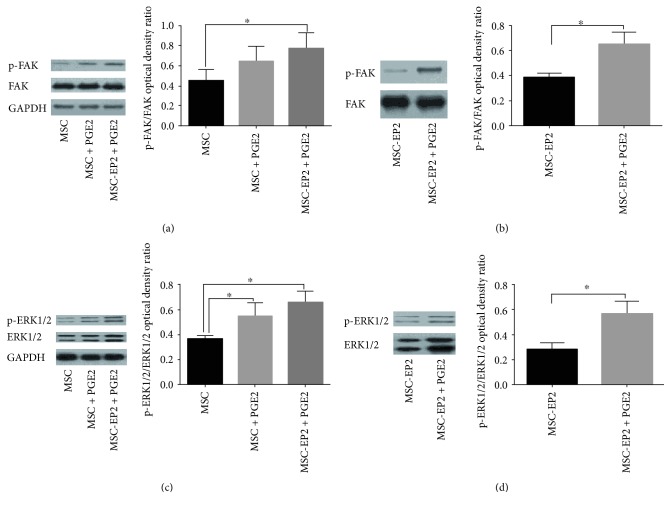
(a) Immunoblotting revealed that the phosphorylated expression level of FAK is much higher in MSCs-EP2 treated with PGE2 compared to the MSCs stimulated with PGE2. (b) Immunoblotting revealed that the phosphorylated expression level of FAK is much higher in PGE2 treated MSCs-EP2 compared to the counterparts without PGE2 stimulation. (c) Immunoblotting revealed that the phosphorylated expression level of ERK1/2 is much higher in MSCs-EP2 treated with PGE2 compared to the MSCs stimulated with PGE2. (d) Immunoblotting revealed that the phosphorylated expression level of ERK1/2 is much higher in PGE2-treated MSCs-EP2 compared to the counterparts without PGE2 stimulation, ^∗^*P* < 0.05.

To investigate whether FAK signaling participated in PGE2-mediated migration of MSCs, the activation status of FAK was assessed by Western blot. Immunoblotting revealed that the phosphorylated expression level of FAK is much higher in PGE2-treated MSCs compared to the counterparts without PGE2 stimulation. We also examine whether EP2 receptor participated in PGE2-induced activation of FAK. The expression of FAK in MSCs-EP2 was also tested. Immunoblotting revealed that the phosphorylated expression level of FAK is much higher in MSCs-EP2 treated with PGE2 compared to the MSCs stimulated with PGE2 (Figures 3(a) and 3(b)).

### 3.7. Involvement of ERK1/2 Pathways in PGE2-Induced MSCs Migration

ERK1/2 plays a crucial role in regulating cell migration via relaying signal from FAK in several cell types. To investigate the involvement of ERK1/2 in PGE2-induced MSCs migration, we detected the effect of PGE2 on ERK1/2 expression. MSCs were stimulated with 1 *μ*mol/L PGE2. MSCs treated with PGE2 showed an increased expression of ERK1/2. We also examine whether EP2 receptor participated in PGE2-induced activation of ERK1/2. The expression of ERK1/2 in MSCs-EP2 was also tested. Immunoblotting revealed that the phosphorylated expression level of ERK1/2 is much higher in MSCs-EP2 treated with PGE2 compared to the MSCs stimulated with PGE2 (Figures 3(c) and 3(d)).

### 3.8. FAK Inhibitor Suppressed PGE2-Induced MSCs Migration

**Figure 4:**
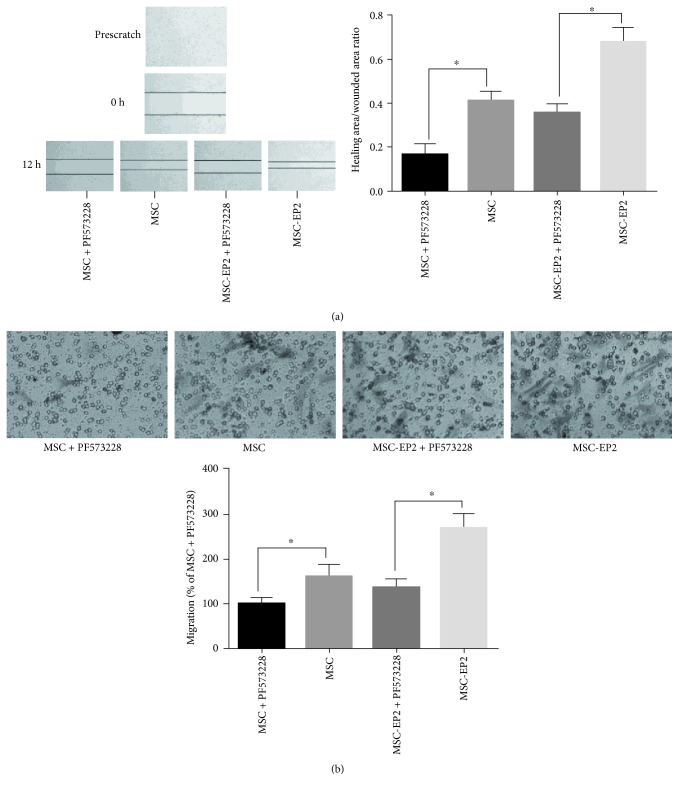
(a) Wound-healing assay demonstrated that MSCs and MSCs-EP2 migration was markedly reduced in the presence of FAK inhibitor PF573228. (b) Transwell assay revealed that the number of FAK inhibitor PF573228 treated MSCs and MSCs-EP2 traversing the membrane was significantly less than the untreated control when incubated with PGE2, ^∗^*P* < 0.05.

To further elucidate the role of FAK in MSCs migration, FAK inhibitor was added at the indicated time. Wound-healing assay demonstrated that MSCs and MSCs-EP2 migration was markedly reduced in the presence of FAK inhibitor PF573228 (Figure 4(a)). Transwell assay revealed that the number of treated MSCs traversing the membrane was significantly less than the untreated control when incubated with PGE2 (Figure 4(b)). Taken together, FAK signaling is a required component for MSCs migration.

### 3.9. ERK1/2 Inhibitor Suppressed PGE2-Induced MSCs Migration

**Figure 5:**
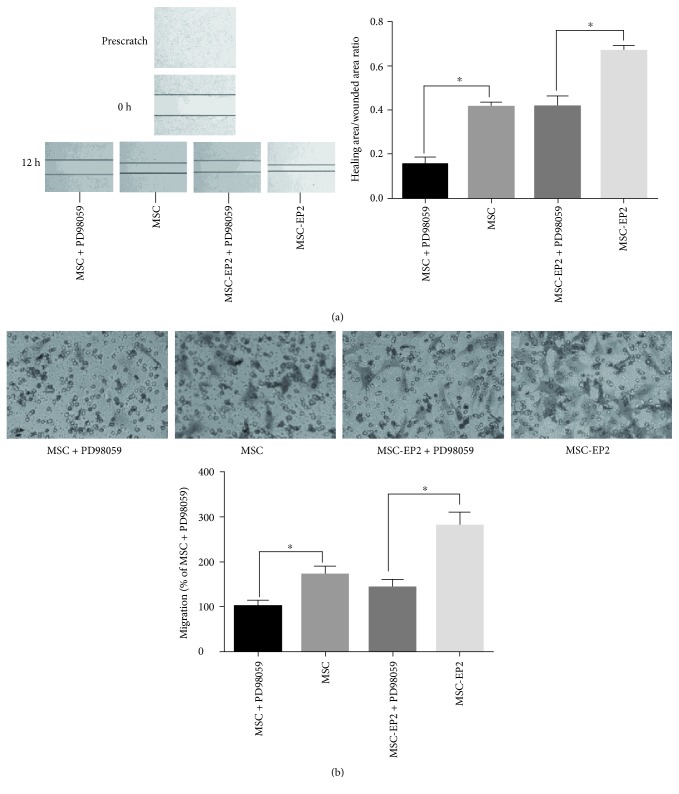
(a) Wound-healing assay demonstrated that MSCs and MSCs-EP2 migration was markedly reduced in the presence of ERK1/2 inhibitor PD98059. (b) Transwell assay revealed that the number of ERK1/2 inhibitor PD98059 treated MSCs and MSCs-EP2 traversing the membrane was significantly less than the untreated control when incubated with PGE2, ^∗^*P* < 0.05.

Furthermore, we confirmed that PGE2-induced ERK1/2 activation was essential for promoting migration and wound healing of MSCs by performing transwell migration assay and wound healing experiment in the presence of PD98059, a specific inhibitor of the ERK1/2 pathway. Inhibition of ERK1/2 by PD98059 also blocked the ability of PGE2 to promote migration of MSCs and fill a wound in in vitro wound-healing assays (Figure 5(a)). In agreement with these findings, Transwell migration assay demonstrated that MSCs and MSCs-EP2 migration was markedly reduced in the presence of PD98059 (Figure 5(b)).

### 3.10. FAK and ERK1/2 Inhibitor Successfully Blocked FAK and ERK1/2, Respectively

**Figure 6:**
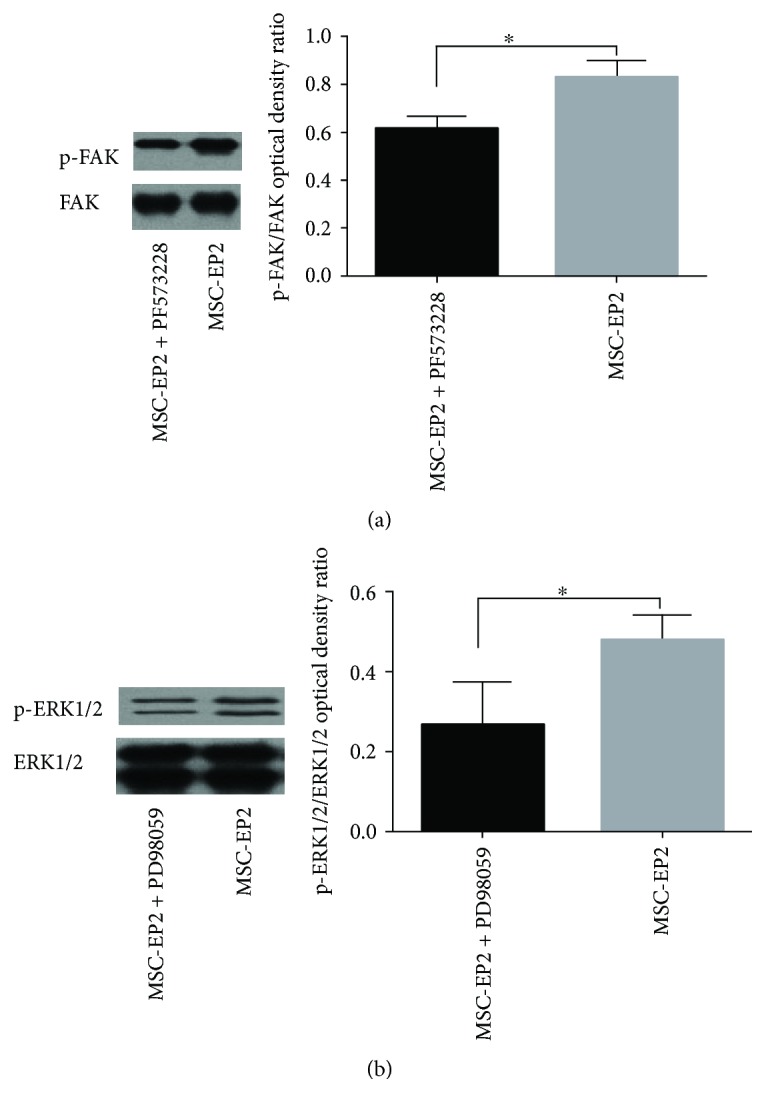
(a) Western blot demonstrated that FAK inhibitor PF573228 successfully blocked FAK. (b) Western blot demonstrated that ERK1/2 inhibitor PD98059 successfully blocked ERK1/2, ^∗^*P* < 0.05.

To demonstrate that the inhibitors successfully blocked FAK and ERK1/2, respectively, we evaluated the activation state of FAK and ERK1/2 following the supplementation of FAK and ERK1/2 inhibitors via Western blot. Our results demonstrated that FAK and ERK1/2 inhibitors successfully blocked FAK and ERK1/2, respectively (Figures 6(a) and 6(b)).

## 4. Discussion

Mesenchymal stem cells have emerged as a leading candidate for treating numerous diseases due to their multipotent capacity, immune privilege properties, and their ability to secrete trophic factors. Beneficial effects induced by MSCs are mainly resulted from their ability to secrete a great variety of paracrine factors rather than the degree of MSC differentiation. Low engraftment of transplanted MSCs in the site of injury is one of the obstacles MSCs face for clinical application [25]. Methods to ensure the sustained expression of cytoprotective cytokines in the injured tissues confer incremental efficacy. A growing body of evidence demonstrated that further increase in the migration ability after MSC delivery produces a pool of trophic factors within the damaged tissue, consequently leading to the enhanced therapeutic outcome [26].

PGE2 plays a critical role in the regulation of inflammation, the modulation of immune process, and the maintenance of intestinal mucosa. COX-derived PGE2 can be produced by many cell types, including macrophage and dendritic cell. The complex roles of PGE2 are mediated by four G-protein coupled receptors (EP1–EP4), delivering different physiologic and pathophysiologic effects. Over the last decade, increasing attention has been paid to the role of PGE2 in the regulation of cellular migration. PGE2 facilitating cellular migration has been well established using several normal and tumor cells [27]. Short-term ex vivo exposure of HSCs to PGE2 was shown to be correlated with increased homing of HSCs after transplantation. Moreover, Donnini et al. have demonstrated that treatment with PGE2 markedly increased the proliferation and migration of squamous carcinoma cells [28].

Prostaglandin E2 (PGE2) is the prominent prostaglandins generated from arachidonic acid by cyclooxygenases and PGE2 sysnthases. PGE2 is a critical mediator that modulates diverse physiologic and pathophysiologic processes via an autocrine or paracrine fashion. PGE2 can potentiate the migratory capacity of MSC to target tissue. A critical step of MSCs therapy is dependent on its ability to migrate into the sites of injury. Further exploration of the underlying molecular mechanism participating in the promigratory ability of PGE2 may provide a novel strategy to improve MSC transplantation efficacy. This may represent a novel strategy to accelerate MSC transplantation efficiency. PGE2/EP2 plays a crucial role in MSC homing to sites of injury. PGE2 serves as a homing signal for MSCs expressing EP2 to navigate MSC to reach the sites of tissue damage. We utilized virus-mediated EP2 transduction to upregulate the expression of EP2 on the surface of MSCs, which resulted in increased migration of MSCs to PGE2 when compared to control MSCs.

Elevated FAK expression was observed after MSCs treated with PGE2. Our results also revealed that FAK inhibitor-treated MSCs exhibited decreased cell motility and a slower wound closure, suggesting that FAK signaling is a critical component in the regulation of MSCs migration. FAK was thought to play a prominent role in the regulation of cell migration, as evidenced in several different cell settings [29]. FAK-null fibroblast exhibit a slower motility as compared with that observed in normal fibroblast. Additionally, carcinoma cells using antisense treatment to reduce FAK expression indicate that these cells exhibit cell motility defect. Conversely, increased cell motility was observed after FAK overexpression in squamous cell. Taken together, FAK may be a required component for MSCs migration similar to that observed in other cell lines.

The downstream signal by which FAK contributes to PGE2-induced MCSs migration was unclear. Extracellular signal-regulated kinase (ERKs) is the subfamily of serine/threonine protein kinases, namely mitogen-activated protein kinases (MAPKs). Two isoforms ERK-1 and Erk-2 have been identified [30]. Erk1/2 has been participating in numerous cell functions, such as cellular proliferation and apoptosis. In particular, evidences have shown that ERK1/2 plays a crucial role in regulating cell migration via relaying signal from FAK in several cell types [31]. The study by Cuevas et al. demonstrated that ERK1/2 was an essential component for fibroblasts migration. Additionally, human osteoblastic cells exhibit reduced cell motility after ERK antisense treatment of these cells [32]. In our study, the expression of ERK1/2 was upregulated in MSCs in response to PGE2 stimulation. Furthermore, exposure of MSCs to specific ERK1/2 inhibitor effectively blocks PGE2-induced migration of MSCs. Taken together, our findings suggested that ERK1/2 signaling plays a key role in regulating of MSC migration.

Several limitations in our study deserve consideration. First, one clear limitation of the current study is that it does not investigate the migration of MSCs over a blood vessel endothelial barrier. Second, pharmacological inhibitors were used to reduce FAK and ERK1/2 expression, deletion of FAK and ERK1/2 by genetic knockdown may make the results more robust. Third, endogenous PGE2 produced from MSCs plays a vital role in inflammatory setting and whether endogenous PGE2 contributes to MSCs migration is still unclear. In addition, evidences indicate that cytoskeletal dynamic was involved in the cellular migration process, and to investigate the change of cellular shape and the formation of stress-fiber may further understand the role of PGE2 in regulating MSCs migration. Thus, future studies are needed to address the above questions.

In conclusion, our findings reveal PGE2 binding to its subtype receptor, EP2 in MSCs, and subsequent activation of FAK and ERK1/2 signaling, thereby facilitating MSCs migration, indicating that activation of EP2 receptor and FAK/ERK pathways may be a promising strategy to accelerate homing efficiency of MSCs, which in turn enhances therapeutic potential of MSCs transplantation.
